# Blue Light Regulates Cell Wall Structure and Carbohydrate Metabolism of Soybean Hypocotyl

**DOI:** 10.3390/ijms24021017

**Published:** 2023-01-05

**Authors:** Chang Wang, Yu Chen, Can Cui, Fuxin Shan, Rui Zhang, Xiaochen Lyu, Lin Lyu, Hanwen Chang, Chao Yan, Chunmei Ma

**Affiliations:** College of Agriculture, Northeast Agricultural University, Harbin 150030, China

**Keywords:** blue light, soybean hypocotyl, cell wall, carbohydrate metabolism, proteome, metabolome

## Abstract

Soybean stem elongation and thickening are related to cell wall composition. Plant morphogenesis can be influenced by blue light, which can regulate cell wall structure and composition, and affect stem growth and development. Here, using proteomics and metabolomics, differentially expressed proteins and metabolites of hypocotyls grown in the dark and under blue light were studied to clarify the effects of blue light on the cell wall structure and carbohydrate metabolism pathway of soybean hypocotyls. Results showed that 1120 differential proteins were upregulated and 797 differential proteins were downregulated under blue light treatment, while 63 differential metabolites were upregulated and 36 differential metabolites were downregulated. Blue light promoted the establishment of cell wall structure and composition by regulating the expression of both the enzymes and metabolites related to cell wall structural composition and nonstructural carbohydrates. Thus, under blue light, the cross-sectional area of the hypocotyl and xylem were larger, the longitudinal length of pith cells was smaller, elongation of the soybean hypocotyl was inhibited, and diameter was increased.

## 1. Introduction

Crop lodging is positively correlated with plant height, internodal length, and stem diameter [1,2,3]. Stem elongation and thickening of crops are associated with cell wall relaxation and material composition [4,5]. Lignin, cellulose, hemicellulose, and pectin are important structural substances of the cell wall that can affect cell wall relaxation and mechanical strength [6,7,8]. In buckwheat, lignin content is closely related to lodging resistance, and a higher level of lignin can result in better lodging resistance [9]. Xue et al. [10] believed that the content of cellulose and hemicellulose in stems decreased with high planting density, and the mechanical strength of maize stems also decreased. Liu et al. [11] also believed that, under intercropping conditions, the content of cellulose and lignin in soybean stems decreased and the stem lodging rate increased. Pectin polysaccharides help to enhance the mechanical strength of the cell wall [12]. The stiffness of pea cotyledons, whose cell walls are rich in galactose, is also significantly enhanced by pectin polysaccharides [13]. Nonstructural carbohydrates, such as sucrose, can promote the formation of cellulose [14,15], thereby increasing stem elasticity and lodging resistance [16]. Ishimaru et al. [17] showed that higher starch content in the stems of rice varieties resulted in stronger stems. The accumulation of carbohydrates increases the stem strength of soybean, while the degradation of nonstructural carbohydrates weakens the stem strength and reduces the lodging resistance index of soybean [5,18]. Shading can lead to the rapid consumption of starch and sucrose in soybean stem tips, significantly reduce the cellulose content in stems, increase the plant height of soybean seedlings, and reduce the lodging resistance of stems [19,20].

As an important signal in plant morphogenesis, blue light can promote stem elongation and thickening of cucumber seedlings [21,22]. However, according to some studies, blue light can inhibit stem elongation and increase the stem diameter of soybean [23,24], pea [25], and cucumber seedlings [26,27]. Blue light can increase the lignin and cellulose content of Arabidopsis seedlings, promote secondary cell wall thickening [28], affect the lignin monomer synthesis pathway, increase the lignin content of Norway spruce needles, and inhibit stem elongation [29]. Moreover, blue light was shown to increase the sucrose and starch contents of upland cotton and rapeseed plantlets and promote stem elongation and thickening of upland cotton, while it inhibited stem elongation and increased the stem diameter of rapeseed plantlets [30,31]. According to He et al. [32], blue light enhanced sucrose transport from leaf to sink and promoted the starch synthesis of potato tuber. Despite the fact that blue light regulates cell wall composition and carbohydrate content, the effects of blue light on soybean cell wall structure and carbohydrate metabolic pathway remain unclear.

The hypocotyl is very sensitive to light [21,26,27,33,34,35]. It is possible to identify differential proteins, metabolites, and related pathways using proteomics and metabolomics [20,36,37,38]. Therefore, in this study, the Chinese soybean plant Heinong 48 was used as experimental material, and the differential expression proteins and metabolites in soybean hypocotyl under blue light treatment were identified and analyzed by combining Tandem mass tag (TMT) quantitative proteomics with non-targeted metabolomics technology. The effects of blue light on the morphological indexes, physiological characteristics, and anatomical structure of soybean hypocotyl were clarified; and the regulation of blue light on the cell wall structure and carbohydrate metabolism pathway of soybean hypocotyl were clarified, thus providing a theoretical basis for light regulation on soybean stem growth.

## 2. Results

### 2.1. Effects of Blue Light on the Anatomical Structure of Soybean Hypocotyls

Table 1 shows the effects of blue light treatment on the length and diameter of soybean hypocotyls. According to the table, blue light treatment significantly reduced hypocotyl length by 63.08% compared with darkness. The hypocotyl diameter under blue light was 17.98% greater than that under darkness.

**Figure 1:**
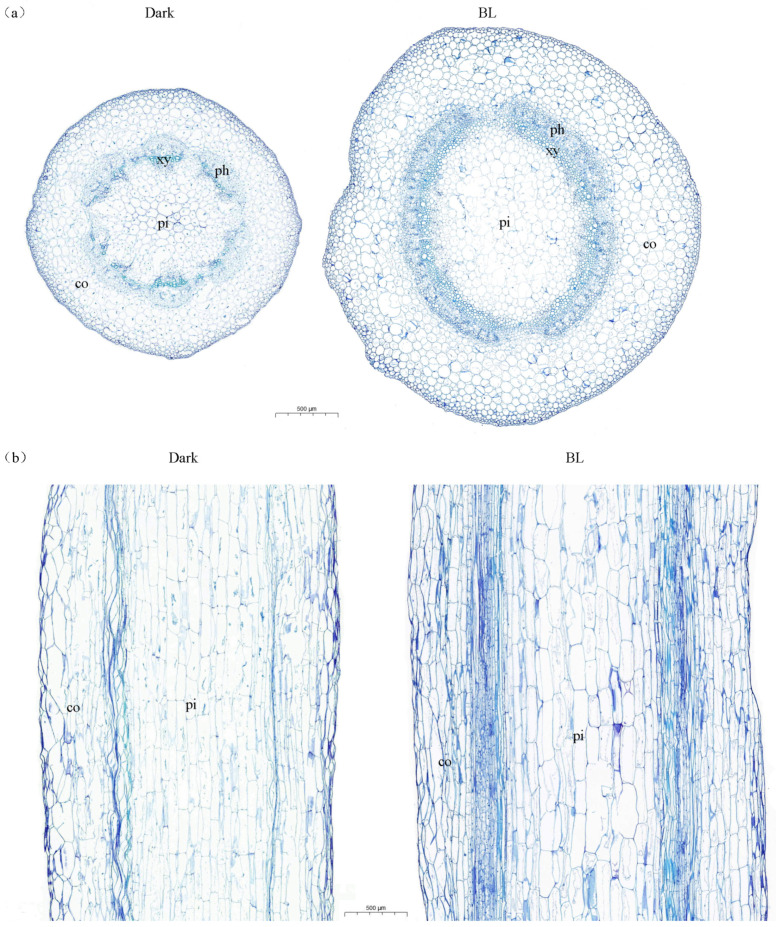
Transverse sections (**a**) and longitudinal sections (**b**) of soybean hypocotyls under dark and blue light treatment. co, cortex; xy, xylem; ph, phloem; pi, pith. Scale bar, 500 μm.

Figure 1 shows the transverse sections (a) and longitudinal sections (b) taken with a 30× optical microscope of soybean hypocotyls grown in darkness and under blue light. The cross-sectional area of hypocotyls under blue light treatment was larger than that of hypocotyls grown in the dark (Figure 1a, Table 2). Furthermore, with blue light treatment, the cortical cells became larger and more regular in shape, the xylem cells increased and became larger, and the proportion of pith area was small (Figure 1a, Table 2). It can be seen from the longitudinal section that in the blue light treatment condition, the longitudinal length of pith cells was smaller, the cortical cells were more regular, and the xylem area was larger (Figure 1b, Table 2).

### 2.2. Effects of Blue Light on the Cell Wall Composition and Carbohydrate Contents of Soybean Hypocotyls

**Figure 2:**
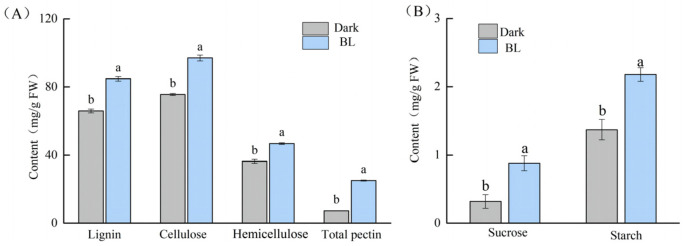
The contents of cell wall composition (**A**) and carbohydrate (**B**) of soybean hypocotyl from plants grown in the dark and under blue light. Values in the table are the mean ± standard error (n = 5). Different letters in the same indicator indicate significant differences between treatments (*p* < 0.05).

Blue light significantly increased the contents of lignin, cellulose, hemicellulose, and total pectin in soybean hypocotyls; which were 28.58%, 28.36%, 28.92%, and 239.72% higher than those in darkness, respectively (Figure 2A). As shown in Figure 2B, blue light significantly increased the contents of sucrose and starch in hypocotyls, which were 175.00% and 59.12% higher, respectively, than those in darkness.

### 2.3. Proteomic Analysis

**Figure 3:**
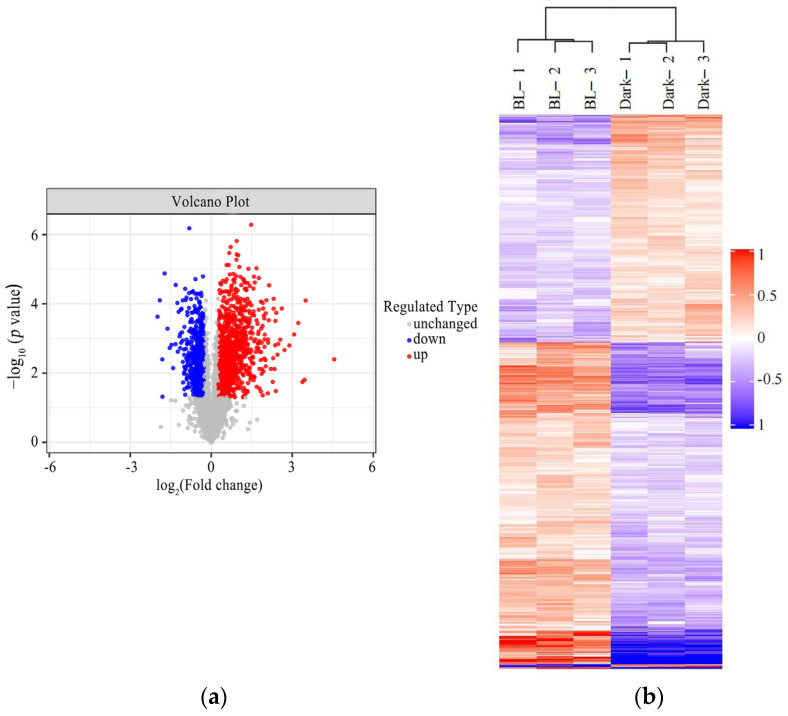
The volcano plot and cluster heatmap of differentially expressed proteins. (**a**) Volcano plot. The abscissa is the fold change (logarithm with base 2), and the ordinate is the significant difference *p* value (logarithm with base 10). Differentially expressed proteins with red dots represent those that have been significantly upregulated, proteins with blue dots represent those that have been significantly downregulated, and proteins with gray dots represent those that have been not changed. (**b**) Cluster heatmap. Each column represents a group of samples (the abscissa is the sample information), each row represents a significantly differentially expressed protein, and the expression levels of the significantly differentially expressed proteins in different samples were normalized by the log_2_ method and displayed in different colors in the heatmap; where red represents significantly upregulated proteins, blue represents significantly downregulated proteins, and gray represents no protein quantification information.

TMT quantitative proteomics were used for proteomic analysis of soybean hypocotyls under the conditions of darkness and blue light by LC–MS/MS data acquisition. A total of 1917 differentially expressed proteins, including 1120 upregulated proteins and 797 downregulated proteins, were screened with fold change > 1.2 times (upregulation greater than 1.2-fold or downregulation less than 0.83-fold) and a *p* value < 0.05 (Figure 3 and Table S1).

**Figure 4:**
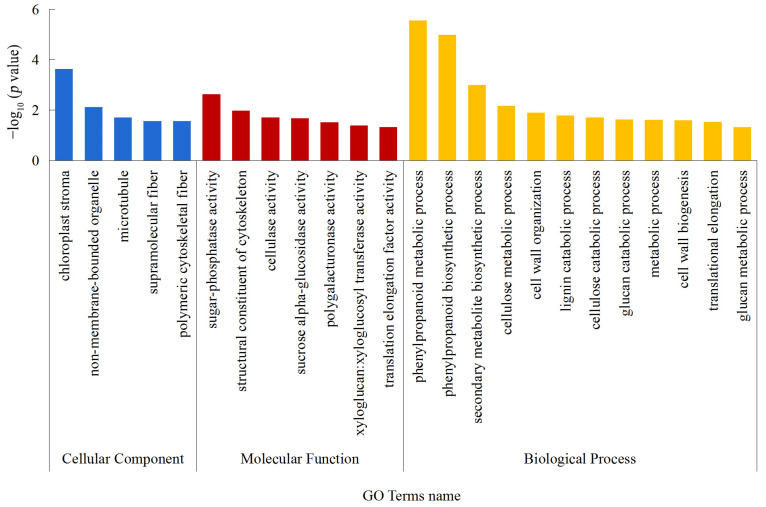
GO annotation statistics of differentially expressed proteins. The abscissa represents GO Level 2 annotation information, including cellular components, molecular functions, and biological processes; which are distinguished by blue, red, and orange, respectively. The ordinate represents the *p* value of each functional category (taking the negative common logarithm, i.e., −log_10_ *p* value).

To understand the functional profile of the differentially expressed proteins, corresponding functional annotations were performed on them through sequence alignment (BLAST) and GO. The differentially expressed proteins were divided into three categories based on their functional characteristics: cellular components (CC), molecular function (MF), and biological process (BP). As shown in Figure 4, in terms of cellular components, a majority of differentially expressed proteins were found in the chloroplast stroma, non-membrane-bounded organelle, microtubule, supramolecular fiber, and polymeric cytoskeletal fiber. In terms of molecular function, the differentially expressed proteins were involved in sugar-phosphatase activity, structural constituent of cytoskeleton, cellulase activity, sucrose alpha-glucosidase activity, polygalacturonase activity, xyloglucan: xyloglucosyl transferase activity and translation elongation factor activity. In terms of biological processes, the differentially expressed proteins were mainly related to phenylpropanoid metabolic process, phenylpropanoid biosynthetic process, secondary metabolite biosynthetic process, cellulose metabolic process, cell wall organization, lignin catabolic process, cellulose catabolic process, glucan catabolic process, metabolic process, cell wall biogenesis, translational elongation, and glucan metabolic process. These results indicated that treatment with blue light mainly affected the process of cell wall relaxation, synthesis and decomposition of structural components, and carbohydrate metabolism.

### 2.4. Metabolome Analysis

To understand the response of soybean hypocotyl metabolites to blue light, ultra-high-performance liquid chromatography-tandem time-of-flight mass spectrometry (UHPLC-Q-TOF MS) was used to analyze the metabolome of soybean hypocotyls under the conditions of darkness and blue light. The differential metabolites were screened according to the criteria OPLS-DA VIP > 1 and *p* value < 0.05, and a total of 99 metabolites were detected, among which 63 were upregulated and 36 were downregulated (Table S2).

**Figure 5:**
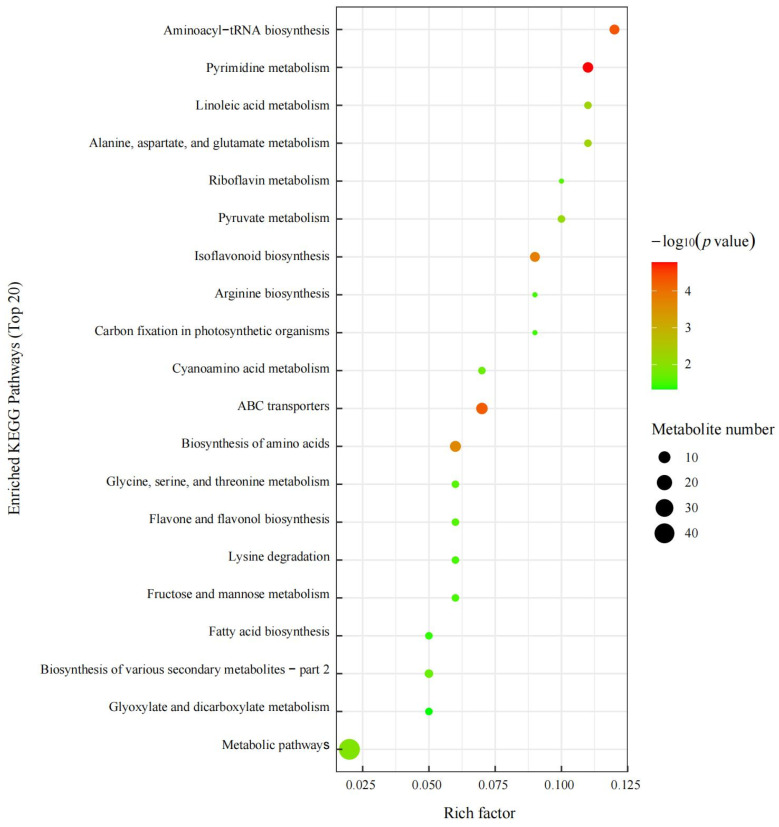
Bubble diagram of the pathways with differential metabolite enrichment. As shown in the bubble diagram, each bubble represents a metabolic pathway (the top 20 metabolic pathways with the highest abundance were selected based on the *p* value). The abscissa and bubble size represent the extent of the pathway’s influence. The larger the bubble is, the greater the influence. In enrichment analysis, the ordinate and color of bubbles represent the *p* value (negative common logarithm, namely, −log_10_ *p* value). The deeper the color is, the smaller the *p* value, and the more obvious the enrichment degree.

Figure 5 shows the bubble diagram of the pathways with differential metabolite enrichment. As seen from the figure, differential metabolites were mainly involved in metabolic pathways, amino acid and carbohydrate metabolism, and biosynthesis of secondary metabolites.

### 2.5. Integrated Proteome and Metabolome Analysis

**Figure 6:**
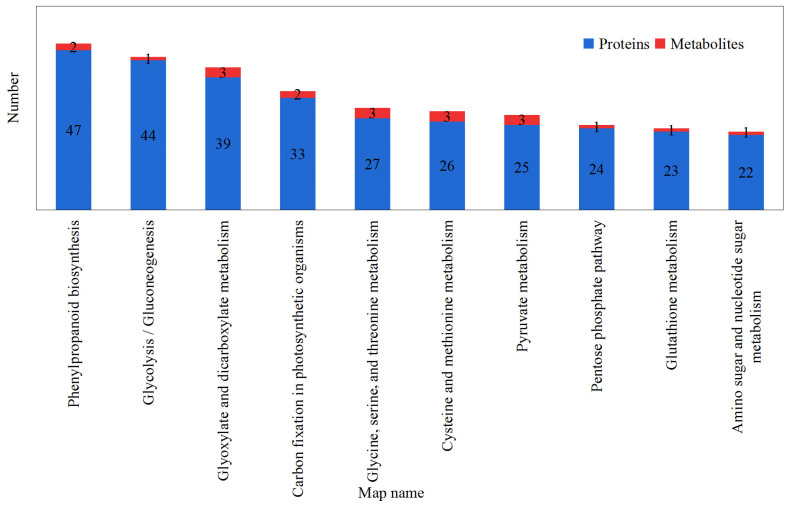
Pathways through which differentially expressed proteins and metabolites participate. The blue color represents differentially expressed proteins, while the red color represents differentially enriched metabolites.

To integrate the pathway data, differential proteins and metabolites were simultaneously projected into the KEGG pathway. Overall, 54 metabolic pathways changed (Table S3). Figure 6 shows the first 10 KEGG pathways implicated in the proteins and metabolites that were identified in this experiment. Differential proteins and metabolites were mainly enriched in phenylpropanoid biosynthesis, glycolysis/gluconeogenesis, glyoxylate and dicarboxylate metabolism, and carbon fixation in photosynthetic organisms, etc. Compared with darkness, the differential proteins and metabolites under blue light treatment were mainly related to phenylpropanoid biosynthesis and carbohydrate metabolism.

## 3. Discussion

### 3.1. Effects of Blue Light on Structural Carbohydrate Metabolism

**Figure 7:**
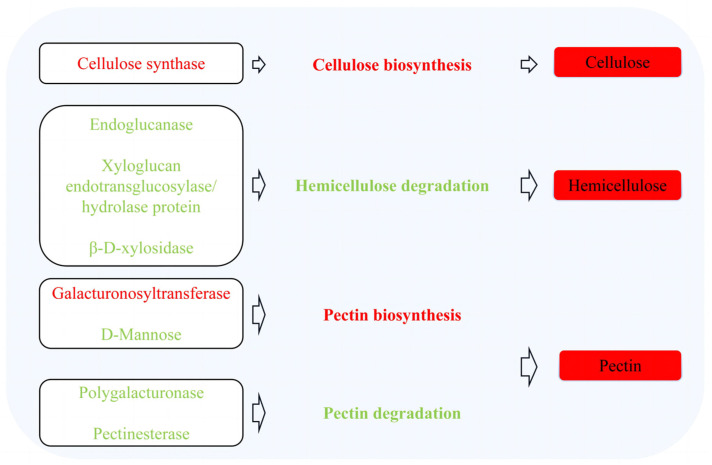
Blue light regulates cellulose, hemicellulose, and pectin metabolism in the hypocotyl. Red text indicates upregulation and green text indicates downregulation. Red solid cubes indicate increased cellulose, hemicellulose, and pectin contents.

Cell wall relaxation and carbohydrate supply are vital to plant cell elongation and expansion [39,40]. Cellulose, hemicellulose, and pectin are the main structural carbohydrates in the plant cell wall, and can improve the mechanical strength of maize stems [10]. Cellulose synthase is related to cell wall cellulose synthesis in *Populus trichocarpa* [41]. Cellulose synthase mutants can lead to reduced cellulose synthesis in Arabidopsis [42]. This study found that the expression of cellulose synthase was upregulated under blue light treatment (Figure 7), and cellulose content was increased (Figure 2). Xyloglucan is an important component of hemicellulose in the cell wall. Endoglucanase (EGase) is involved in the hydrolysis of xyloglucan [43], affecting the cell wall relaxation of *Arabidopsis thaliana* [44] and promoting the elongation of cotton fiber cells by breaking the endo-1,4-β bond of cellulose-xylan [45]. The effect of xyloglucan endotransglucosylase/hydrolase (XTH) of the xyloglucan-cellulose network on cell wall relaxation of onion (*Allium cepa*) bulb scales has been studied previously [46]. The upregulation of XTH expression can promote relaxation of the cell wall of soybean stems, affect internode elongation [36], and promote elongation growth of petioles in Arabidopsis [47]. β-xylosidase plays a key role in hemicellulose degradation [48]. In this study, the expression levels of endoglucanase, XTHs and β-D-xylosidase were all downregulated under blue light treatment (Figure 7), indicating that blue light can inhibit the hydrolysis of xyloglucan, reduce the activity of XTHs, inhibit cell wall relaxation, and reduce hemicellulose degradation by inhibiting the activity of β-D-xylosidase. Mannose is an important sugar chain of mannan and an important component of hemicellulose [49]. According to the present study, blue light treatment reduced D-mannose (Figure 7) but increased the content of hemicellulose (Figure 2). According to previous studies, hemicellulose is a polymer composed of many different types of monosaccharides [50]; and the content of sugars other than mannan may increase, which in turn enhances the content of hemicellulose. Galacturonosyltransferase (GAUT) is mainly involved in pectin and xylan biosynthesis [51,52], while polygalacturonase (PG) and pectinesterase (PE) are important enzymes for the degradation of plant pectin skeleton structure [53,54]. Sterling et al. [55] suggested that several GAUT1 genes encoding GAUT in Arabidopsis were related to pectin synthesis. A study by [56] found that PG activity and pectin degradation activity increased during the late softening stage of melon fruit. In this study, blue light treatment led to the upregulation of GAUT, while the expression of PG and PE was downregulated (Figure 7); indicating that blue light could increase pectin content (Figure 2) and reduce pectin degradation. In our previous studies on the regulation of red light on soybean hypocotyl elongation, it was found that red light promoted the expression of enzymes related to the conversion of serine and threonine to glycine, decreased the level of intracellular reactive oxygen species (ROS), reduced the degradation of cell wall polysaccharides, and increased cellulose and hemicellulose content in soybean hypocotyls; thereby inhibiting soybean hypocotyl elongation [57]. The difference in this study is that blue light affects the cell wall structural carbohydrate metabolism process by affecting the expression of enzymes involved in the synthesis and decomposition of cellulose, hemicellulose, and pectin; thereby affecting the cell wall structure and accumulation of components, and thus inhibiting soybean hypocotyl elongation.

### 3.2. Effects of Blue Light on Nonstructural Carbohydrate Metabolism 

**Figure 8:**
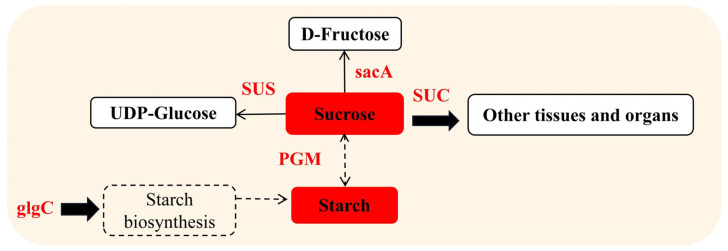
Blue light regulates sucrose and starch concentrations and related enzyme activities in the hypocotyl. SacA, β-fructofuranosidase; SUS, sucrose synthase; SUC, sucrose transport protein; PGM, phosphoglucomutase; glgC, glucose-1-phosphoadenosyltransferase. The red solid squares represent the increased concentrations of sucrose and starch. The red text represents upregulated protein expression.

Sucrose is the initial substrate for cellulose and starch synthesis. Sucrose synthase (SUS) can degrade sucrose in cotton fiber [58], which provides a direct substrate for cellulose synthesis and uridine diphosphate glucose and improves the rate of cellulose deposition [59]. β-Fructofuranosidase (sacA) can catalyze the irreversible hydrolysis of sucrose to fructose and glucose [60]. Sucrose transport protein (SUC) affects the distribution of sucrose to other tissues and organs. The sucrose transport of Arabidopsis SUC2 mutant plants is blocked and plant development is hindered [61]. In this study, the expression of sucrose synthase, acid β-fructofuranosidase, and sucrose transport protein was upregulated under blue light (Figure 8); indicating that blue light can promote cellulose synthesis by regulating the decomposition and transport of sucrose. Glucose-1-phosphoadenosyltransferase (glgC) is an important regulator of starch synthesis. Upregulation of glgC in corn, rice, and wheat can increase the starch content in crops [62]. Phosphoglucomutase (PGM) is involved in the conversion of sucrose and starch; and antisense inhibition of PGM activity inhibits sucrose synthesis in potato leaves and reduces starch levels in tubers [63]. It was shown that the expression of PGM was upregulated under blue light treatment and the contents of sucrose and starch in hypocotyls were increased in this study (Figure 2); thus indicating that blue light could promote the synthesis of carbohydrates, as shown by Wang et al. [64] in cucumber leaves.

### 3.3. Effects of Blue Light on Lignin Synthesis

**Figure 9:**
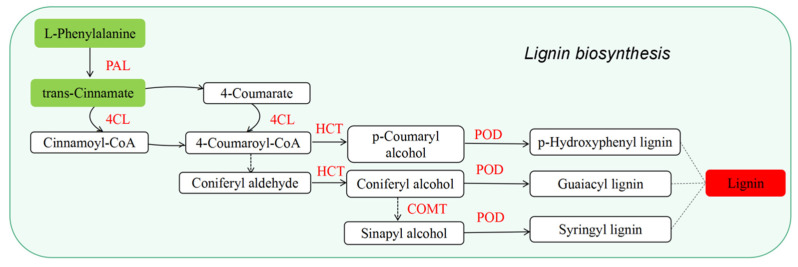
Regulation of blue light on the lignin synthesis pathway. PAL, phenylalanine ammonia-lyase; 4CL, 4-coumarate-CoA ligase; HCT, spermidine hydroxycinnamoyl transferase; COMT, caffeic acid 3-O-methyltransferase; POD, peroxidase. The red solid block indicates that lignin content was increased under the blue light; and the green solid block indicates that the expression of differential metabolites was decreased. Red text indicates upregulation of protein expression.

Lignin is an important factor affecting cell wall strength, which can provide strong mechanical support for soybean stems [65]. Lignin synthesis starts from phenylalanine and finally polymerizes into lignin through a series of enzyme catalyses [66]. As the first step in the biosynthesis of phenylpropanoid, phenylalanine ammonia-lyase (PAL) plays a pivotal role [67]. The lignin content of Arabidopsis PAL gene double mutant plants was significantly reduced [68], although blue light can upregulate the expression of the PAL gene in the needles of Norway spruce [29]. Wang et al. [69] suggested that oat varieties with high activity of proteins such as PAL, 4CL, and CAD have high lignin content and strong lodging resistance. Nguyen et al. [70] believed that several genes were involved in the accumulation of lignin in wheat stems, such as 4CL, HCT, COMT, and others. Our previous study on red light regulation of soybean hypocotyl elongation found that red light treatment upregulated key enzymes in the lignin synthesis pathway―including PAL, POD, COMT, and 4CL―and promoted lignin synthesis [57]. The present study was consistent with the red light study, which showed that PAL, 4CL, HCT, POD, and COMT were all upregulated by blue light treatment (Figure 9). It is suggested that blue light may promote lignin monomer polymerization, increase lignin content by regulating these key enzymes involved in the regulation of phenylpropanoids in hypocotyls (Figure 2), enhance cell wall firmness, and inhibit hypocotyl elongation; moreover, its regulatory mechanism is similar to that of red light [57]. This study also found that expression levels of L-phenylalanine and trans-cinnamate were both downregulated under blue light conditions. The analysis showed that PAL and 4CL were upregulated during the conversion of L-phenylalanine to trans-cinnamate and the conversion of trans-cinnamate to cinnamoyl-CoA, which may accelerate the consumption of L-phenylalanine and trans-cinnamate.

## 4. Materials and Methods

### 4.1. Experimental Design

We conducted the experiment in 2020 in an enclosed light chamber (120 cm × 60 cm × 180 cm in length × width × height). The soybean variety Heinong 48 was used as experimental material, which was bred by the Soybean Research Institute of the Heilongjiang Academy of Agricultural Sciences. Several soybean seeds of similar size and shape were selected and sown in three holes in a pot (9 cm diameter, 10 cm height), two seeds per hole. The pots were eventually refilled with only one seedling each. We cultured all pots in darkness at 25 °C, after sowing until hypocotyls grew approximately 5 cm above the soil surface. Two groups, one treated with darkness and the other with monochromatic blue light, were prepared in the test. Blue light was generated by a 12-watt LED lamp with a uniform arrangement of bulbs. The blue LED lamp has a wavelength of 455–465 nm, and begins to irradiate continuously for 24 h in the hypocotyl length of about 5 cm. We placed the light source 1 cm from the seedling stem on only one side, to irradiate the hypocotyl vertically. The photon flux density was 45 ± 0.03 μmol·m^−2^·s^−1^. During the third day of light treatment, the soybean hypocotyl diameter and length were measured. At the same time, the anatomical structure and material components, as well as the nonstructural carbohydrates, proteome, and metabolism were analyzed, and 18 replicates were set for each treatment. All of the experiments were carried out in an incubator controlled at 25 ± 1 °C with a relative humidity of 50 ± 5%.

### 4.2. Anatomical Structure Sampling and Observation

The hypocotyls of five soybean plants with the same growth in each group were cut into small pieces (5 mm across) after three days of blue light treatment. The cut samples were fixed in a brown bottle containing FAA fixative solution. The samples were pumped into the bottom of the sample by a vacuum pump; dehydrated and made transparent; and then a drop of toluidine blue was added, followed by paraffin embedding, slicing (10 μm thickness), patching, baking, wax removal, dyeing, and sealing. Finally, in order to observe the slices, an upright optical microscope (Nikon Eclipse E100, Tokyo, Japan) was used; and a digital imaging system (Nikon DS-U3, Tokyo, Japan) was used to take photos in the slices. The length of longitudinally sectioned pith cells was measured by randomly selecting 60 pith cells from 10 fields of view. Area of pith cell, cross-sectional diameter, cross-sectional area, and the ratio of pith cell area to cross-sectional area were measured in a cross-sectional map, repeated 10 times. 

### 4.3. Determination of Cell Wall Composition and Carbohydrate Contents

The hypocotyls of five soybean plants with the same growth status were selected for sampling following a three-day blue light treatment, wrapped in tin paper, rapidly frozen in liquid nitrogen, and then transferred to a freezer at −80 °C for preservation. An enzyme-linked immunosorbent assay (ELISA) was used to analyze lignin (Product number ml076959), cellulose (Product number ml607720), and hemicellulose (Product number ml077332) in 0.1 g fresh hypocotyl samples with a kit (Shanghai Enzyme-linked Biotechnology Co., Ltd., Shanghai, China). A kit from Beijing Solarbio Science & Technology Co., Ltd. (Beijing, China) was used to determine the contents of total pectin (Product number BC1405), starch (Product number BC0705), and sucrose (Product number BC2465). The above tests were performed five times for each sample.

### 4.4. Proteomic Analysis

After three days of light treatment, 3 g of soybean hypocotyls were taken, divided into three portions, washed with PBS, wrapped in tinfoil, frozen in liquid nitrogen, and then stored at −80 °C for proteomics analysis; this process was performed three times per condition. Proteome analysis was performed according to the method of Wang et al. [57]. First, protein extraction, peptide digestion, and peptide quantification were carried out. The peptides were labeled according to the instructions of the TMT labeling kit (Thermo Fisher Scientific, Waltham, MA, USA). A high-pH reversed-phase peptide fractionation kit and AKTA Purifier 100 were used for RP and SCX fractionation. The fractionation method and parameter information are as described by Wang et al. [57]. LC-MS/MS data acquisition was then performed, and each sample was separated using a Q Exactive mass spectrometer (Thermo Fisher Scientific, Waltham, MA, USA) coupled to an Easy nLC (Proxeon Biosystems, now Thermo Fisher Scientific, Waltham, MA, USA) [57]. A Q-Exactive mass spectrometer was used to analyze the samples after chromatographic separation. The specific parameters of mass spectrometry were described by Wang et al. [57]. The MASCOT engine (Matrix Science, London, UK; version 2.2) in Proteome Discoverer 1.4 software was used for identifying and quantifying proteins using “Glycine_max” from the NCBI database.

### 4.5. Metabolomic Analysis

After three days of light treatment, 8 g of soybean hypocotyls were taken and divided into 8 portions, washed with PBS, wrapped in tinfoil, frozen in liquid nitrogen, and then stored at −80 °C for subsequent metabolomic analysis. For metabolomic analysis, eight replicates were set, and the method of Wang et al. [57] was used. First, the hypocotyl samples were dissolved in 1 mL precooled methanol/acetonitrile/water solution (2:2:1, *v*/*v*/*v*). After vortexing, low-temperature ultrasound, centrifugation, and vacuum drying, 100 μL acetonitrile aqueous solution (acetonitrile: water = 1:1, *v*/*v*) was redissolved for mass spectrometry analysis. The supernatant was analyzed after vortexing and centrifugation. Next, an Agilent 1290 Infinity LC ultrahigh-performance liquid chromatography (UHPLC) HILIC column was used for separation, and an AB Triple TOF 6600 mass spectrometer was used to collect the primary and secondary spectra of the samples. The parameters were as described by Wang et al. [57]. In order to minimize the effect of instrument detection signal fluctuations, samples were analyzed continuously and based on a random process. To monitor and evaluate the stability of the system and the reliability of the experimental data, QC samples were added to the sample queue.

### 4.6. Statistical Analysis

Data analysis and graphs were performed using SPSS 21.0 and Origin 9.0 software. The proteins with significantly different expression were screened according to the fold change >1.2 times (upregulation by more than 1.2 fold or downregulation of less than 0.83 fold) and *p* value < 0.05 (T test). The metabolites with significantly different concentrations were screened according to the criteria of satisfying both OPLS-DA VIP > 1 and *p* value < 0.05 [57]. A Gene Ontology (GO) annotation was performed on the target protein set using Blast2GO. Based on the KEGG database information, KEGG pathway annotation and enrichment analysis of differential metabolites were performed. A combination of KEGG annotations and enrichment information was integrated using R software (version 3.5.1) [57].

## 5. Conclusions

Blue light promoted the establishment of cell wall structure and composition by regulating enzymes and metabolites related to cell wall structural composition and nonstructural carbohydrates. Thus, with blue light, the cross-sectional area of the hypocotyl and xylem were larger, the longitudinal length of pith cells was smaller, the elongation of soybean hypocotyl was inhibited, and the diameter was increased.

## Figures and Tables

**Table 1:** Effects of blue light on hypocotyl length and diameter.

Treatments	Length (cm)	Diameter (mm)
Dark	16.25 ± 0.66a	2.67 ± 0.01b
BL	6.00 ± 0.00b	3.15 ± 0.12a

Values in the table are the mean ± standard error (n = 18). Different letters in the same column indicate significant differences between treatments (*p* < 0.05). BL: Blue light. The same below.

**Table 2:** Parameters related to anatomical structure of soybean hypocotyls under blue light.

Treatments	Length of Longitudinal Pith Cells (mm)	Area of Pith Cell (mm^2^)	Cross-Sectional Diameter (mm)	Cross-Sectional Area (mm^2^)	Ratio of Pith Cell Area to Cross-Sectional Area (%)
Dark	0.37 ± 0.01a	0.98 ± 0.05b	2.12 ± 0.01b	3.55 ± 0.08b	27.36 ± 1.13a
BL	0.34 ± 0.01b	1.53 ± 0.11a	3.09 ± 0.03a	7.32 ± 0.23a	20.71 ± 0.84b

Values in the table are the mean ± standard error. The length of longitudinally sectioned pith cells was measured by randomly selecting 60 pith cells from 10 fields of view. Area of pith cell, cross-sectional diameter, cross-sectional area, and the ratio of pith cell area to cross-sectional area were measured in a cross-sectional map, repeated 10 times. Different letters in the same column indicate significant differences between treatments (*p* < 0.05). BL: Blue light.

## Data Availability

The data presented in this study are available on request from the corresponding author.

## Supplementary Materials

The following are available online at https://www.mdpi.com/article/10.3390/ijms24021017/s1.
